# Major Co-localized QTL for Plant Height, Branch Initiation Height, Stem Diameter, and Flowering Time in an Alien Introgression Derived *Brassica napus* DH Population

**DOI:** 10.3389/fpls.2018.00390

**Published:** 2018-03-28

**Authors:** Yusen Shen, Yang Xiang, Ensheng Xu, Xianhong Ge, Zaiyun Li

**Affiliations:** ^1^National Key Laboratory of Crop Genetic Improvement, National Center of Oil Crop Improvement, College of Plant Science and Technology, Huazhong Agricultural University, Wuhan, China; ^2^Guizhou Rapeseed Institute, Guizhou Academy of Agricultural Sciences, Guiyang, China

**Keywords:** *Brassica napus*, alien introgression, QTL cluster, stem-related trait, candidate gene

## Abstract

Plant height (PH), branch initiation height (BIH), and stem diameter (SD) are three stem-related traits that play crucial roles in plant architecture and lodging resistance. Herein, we show one doubled haploid (DH) population obtained from a cross between Y689 (one *Capsella bursa*-*pastoris* derived *Brassica napus* intertribal introgression) and Westar (*B*. *napus* cultivar) that these traits were significantly positively correlated with one another and with flowering time (FT). Based on a high-density SNP map, a total of 102 additive quantitative trait loci (QTL) were identified across six environments. Seventy-two consensus QTL and 49 unique QTL were identified using a two-round strategy of QTL meta-analysis. Notably, a total of 19 major QTL, including 11 novel ones, were detected for these traits, which comprised two QTL clusters on chromosomes A02 and A07. Conditional QTL mapping was performed to preliminarily evaluate the genetic basis (pleiotropy or tight linkage) of the co-localized QTL. In addition, QTL by environment interactions (QEI) mapping was performed to verify the additive QTL and estimate the QEI effect. In the genomic regions of all major QTL, orthologs of the genes involved in phytohormone biosynthesis, phytohormone signaling, flower development, and cell differentiation in *Arabidopsis* were proposed as candidate genes. Of these, *BnaA02g02560*, an ortholog of *Arabidopsis GASA4*, was suggested as a candidate gene for PH, SD, and FT; and *BnaA02g08490*, an ortholog of *Arabidopsis GNL*, was associated with PH, BIH and FT. These results provide useful information for further genetic studies on stem-related traits and plant growth adaptation.

## Introduction

Rapeseed (*Brassica napus* L., AACC, 2n = 38) is the second largest oil crop in the world after soybean, with an annual production of ~70 million metric tons in recent years (2015–2017) (https://apps.fas.usda.gov/psdonline/psdReport.aspx). Plant height (PH), branch initiation height (BIH), and stem diameter (SD) are stem-related traits that affect plant architecture in rapeseed (Cai et al., 2016). Together with flowering time (FT), these traits constitute selection targets in the breeding of varieties with preferred morphologies and adaptations.

Both PH and FT are complex traits that are controlled by endogenous and environmental factors (Zhang et al., 2015). Extensive research has shown that many phytohormones, including gibberellins (GAs), brassinosteroids (BRs), auxin (IAA), and strigolactones (SLs), participate in PH regulation. Genes associated with phytohormone biosynthesis and signaling ultimately determine plant height (Fujioka and Yokota, 2003; Sakamoto, 2004; Muhr et al., 2016; Zhao et al., 2017; Li et al., 2018). Using *Arabidopsis thaliana* as a model for long-day species and rice (*Oryza sativa*) for short-day species, remarkable progress has been made in identifying FT regulators (Blümel et al., 2015). Five major pathways (Srikanth and Schmid, 2011) and approximately 180 genes have been found in *A. thaliana* (Fornara et al., 2010). For example, CONSTANS (CO) was considered to be a network hub integrating internal and external signals into the photoperiodic flowering pathway (Shim et al., 2017). Several lines of evidence indicate that CO activates *FLOWERING LOCUS T* (*FT*) and *TWIN SISTER OF FT* (*TSF*) (Tiwari et al., 2010; Pearce et al., 2017; You et al., 2017). In turn, *FT* and *TSF* encode a long- range signal (florigen) that carries information about the inducement of flowering from the leaf to the shoot apical meristem (SAM) (You et al., 2017).

Quantitative trait loci (QTL) mapping is a powerful tool for elucidating the genetic architecture of complex traits (Mauricio, 2001), and many QTL for PH and FT have been identified in *B*. *napus* (Raman et al., 2013; Luo et al., 2014; Wang et al., 2015; Liu et al., 2016; Sun et al., 2016), including several co-localized QTL in biparental populations (Quijada et al., 2006; Udall et al., 2006; Mei et al., 2009). In contrast, QTL associated with BIH and SD are comparatively rare. Recently, significant SNPs relating to BIH and PH were detected via a genome-wide association study (GWAS), in which it was also found that BIH was significantly correlated with PH in this natural population (Zheng et al., 2017). In the case of co-localized QTL or SNPs for different traits, it may be that such clustering is due to either pleiotropy or linkage (Quijada et al., 2006). To distinguish linkage from pleiotropy, conditional QTL mapping method was proposed to evaluate the genetic relationships between correlated traits at the QTL level (Zhu, 1995; Wen and Zhu, 2005).

In the present study, QTL mapping for PH, BIH, SD, and FT were performed, based on a high-density SNP map constructed from a DH population developed from a cross involving one *Capsella bursa*-*pastoris* derived *B. napus* intertribal introgression line with compressed branches and wooden stems (Shen et al., 2018). Additive QTL and the QTL by environment interactions (QEI) were detected across multiple environments. Notably, 19 major QTL, 11 of which had not been previously identified, were revealed on chromosomes A02 and A07, and organized into two QTL clusters. We then analyzed the genetic basis (pleiotropy or tight linkage) of the co-localized QTL using conditional QTL mapping. In addition, candidate genes within the confidence interval of the major QTL were proposed based on the known function of *Arabidopsis* orthologs. These results enabled us to better understand the genetic mechanisms of the four traits and facilitate marker-based breeding to improve plant architecture and growth adaptation, and ultimately to increase rapeseed yield.

## Materials and methods

### Plant material and growth conditions

A *B. napus* YW-DH population encompassing 208 lines was used as a mapping population (Shen et al., 2018). The parents of this doubled-haploid (DH) population were *B. napus* introgression Y689 and cultivar Westar. Y689 was derived from a partial intertribal hybrid between *B. napus* cv. Zhongyou 821 and *Capsella bursa-pastoris* (L.) Medic (2n = 4x = 32) (Chen et al., 2007; Zhang et al., 2013; Shen et al., 2018). Westar is a spring-type canola that is widely used in experiments involving the role of genetic architecture in resistance to *Sclerotinia sclerotiorum* and timing of flowering (Zhao et al., 2009; Nelson et al., 2014). Y689 is both taller and flowers later than Westar.

The DH lines and the two parents were grown using a randomized complete block design with two replicates. Each plot contained two rows with 30 cm row spacing and 20 cm spacing between plants. Year-location combinations were treated as environments, which were coded as 15WH (Wuhan, Hubei Province, China, 2015-2016), 15CD (Chengdu, Sichuan Province, China, 2015-2016), 15ER (Ezhou, Hubei Province, China, 2015-2016), 16WH (Wuhan, Hubei Province, China, 2016-2017), 16XN (Xining, Qinghai Province, China, 2016) and 17WN (Weining, Guizhou Province, China, 2017). The areas WH, CD, and ER are semi-winter-type rapeseed growing areas, in which seeds are typically sown in October and plants are harvested in May of the coming year, whereas the areas XN and WN are spring-type rapeseed growing areas, in which sowing typically occurs in May and plants are harvested in September. Detailed information about the six environments is listed in Table S1. The phenotypes of PH, BIH, SD, and FT were measured in six, five, five, and five of the environments, respectively (Table S1). Normal agronomic practices for rapeseed were conducted in field management.

### Phenotypic evaluations and statistical analysis

Flowering time was recorded for each plot when half of the plants in the plot initiated flowering, and the days from the sowing date to the flowering time were used for the phenotype. At maturity, five representative plants from the center of each plot were selected for measurements of PH, BIH, and SD. Plant height was defined as the distance from the base of the above-ground plant to the tip of the main inflorescence; BIH was defined as the distance from the base of the above-ground plant to the first primary branch with siliques; and SD was defined as the stem thickness at 20 cm above the soil surface using slide calipers.

For PH, BIH, and SD, data from the five plants were averaged to represent the phenotype of a plot, and data from replicated plots were averaged to represent the phenotype of a DH line. Broad-sense heritability (*h*^2^) and correlations among the four traits were analyzed using SAS GLM and CORR packages, respectively (Shen et al., 2018; SAS v9.3, SAS Institute, Cary, NC, USA).

### QTL mapping

Based on a YW-DH population deriving from a cross between an alien introgression line Y689 and *B. napus* cv. Westar, we constructed a linkage map using 3,073 available SNP markers, which covered a length of 2,242.14 cM and had an average marker interval of 0.73 cM (Shen et al., 2018). After the phenotypic investigation across six environments for PH and five environments for BIH, SD, and FT, QTL analysis was carried out for each experiment independently using Windows QTL Cartographer 2.5 with the composite interval mapping (CIM) method (Wang et al., 2012). Likelihood of odd (LOD) thresholds for claiming QTL for PH, BIH, SD, and FT were determined by 1,000 permutations at *P* = 0.05. All additive QTL for the four traits were analyzed by two-round QTL meta-analysis (Shi et al., 2009), with BioMercator 4.2 software (Sosnowski et al., 2012). In the first round, significant additive QTL for each trait were integrated into the consensus QTL. In the second round, the consensus QTL detected from different traits were integrated into unique QTL. In addition, consensus QTL explained more than 15% of the phenotypic variation (PVE) in one environment or with PVEs higher than 10% in two or more environments defined as major QTL.

To dissect the genetic basis (pleiotropy or tight linkage) of the co-localized QTL, conditional QTL mapping was performed (Li et al., 2014; Zhang et al., 2016). Conditional phenotypic values y (T1|T2) were obtained using the software QGAStation 2.0 (http://ibi.zju.edu.cn/software/qga), where T1 and T2 represent the target traits; T1|T2 signifies that T1 was conditioned on T2, indicating that y (T1|T2) were the conditional values obtained when the effect of T2 on T1 was removed. The conditional values were then used to scan QTL via the same method as was used for unconditional QTL mapping described above.

To detect the QTL by environment interactions (QEI), QTL analysis was also carried out using QTL IciMapping 4.1 (http://www.isbreeding.net) with the inclusive composite interval mapping (ICIM) method. The ICIM-MET functionality in the software performed a stepwise regression to identify the most significant markers at 0.001 probability level with the step size of 1 cM. The LOD thresholds of QTL were determined by a 1,000 permutation test at a 95% confidence level. To distinguish from the QTL identified by CIM, we designated the QTL identified by ICIM as “combined QTL.”

Following the previously described nomenclature (Udall et al., 2006), unconditional additive QTL were designated with an initial letter “q” followed by the abbreviation of trait name, and the detected QTL order on the chromosome (e.g., qPH.A01-1). Consensus QTL, unique QTL, combined QTL, and conditional additive QTL were thus designated with the initial letters “cq-,” “uq-,” “Iq-,” and “cdq-” (e.g., cqPH.A02-1, uqPH.C01-1, IqPH.A01-2, cdqPH|FT.C01-2), respectively.

## Results

### Phenotypic performances of the parental and DH lines

**Figure 1:**
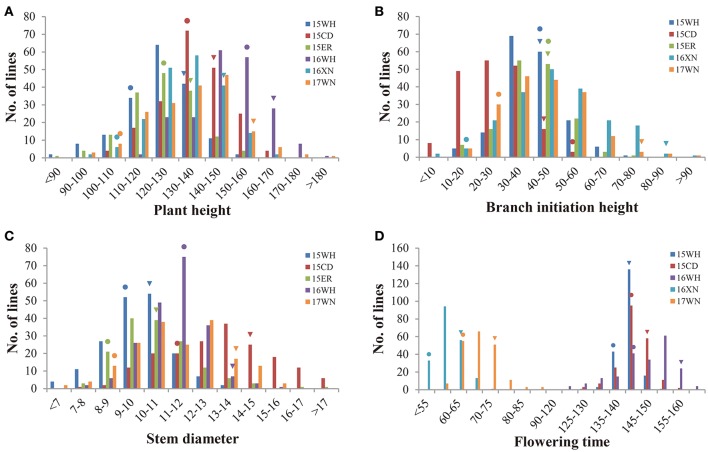
Phenotypic distribution of PH, BIH, SD, and FT in the YW-DH population derived from the cross “Y689” × “Westar.” **(A)** Plant height (PH). **(B)** Branch initiation height (BIH). **(C)** Stem diameter (SD). **(D)** Flowering time (FT). 15WH, 15CD, 15ER, 16WH, 16XN, and 17WN represent six environments with different colors. 15WH: Wuhan, 2015–2016; 15CD: Chengdu, 2015–2016; 15ER: Ezhou, 2015–2016; 16WH: Wuhan, 2016–2017; 16XN: Xining, 2016; 17WN: Weining, 2017. The triangles and rounds indicate “Y689” and “Westar,” respectively.

Parents of Y689 and Westar differed significantly in PH, SD, and FT in all experimental environments, whereas for BIH, significant differences were only detected between the spring-type rapeseed growing areas of 16XN and 17WN. Descriptive statistics of these four traits for the two parents, as well as the DH lines across multiple environments, are listed in Table 1. The DH lines exhibited broad variations among the four traits. Frequency distributions of the phenotypic values of the four traits in the DH lines are shown in Figure 1. Among these traits, FT was differed significantly between spring-type and semi-winter-type rapeseed areas, with averages ranging from 58.7 ± 3.9 to 68.7 ± 5.3 and 142.0 ± 3.0 to 146.7 ± 8.6, respectively (Table 1 and Figure 1).

**Table 1:** Statistical analysis of PH, BIH, SD, and FT for the DH lines and their parents.

**Trait^a^**	**Environment^b^**	**Parents**		**DH lines**				
		**Y689**	**Westar**	**Range**	**Mean ± SD**	**Skewness**	**Kurtosis**	**Heritability**
PH	15WH	132.40 ± 8.04	113.86 ± 9.96^***^^c^	81.70–153.70	123.66 ± 12.80	−0.62	0.53	0.78
	15CD	147.38 ± 13.75	131.38 ± 7.93^***^	104.40–167.10	137.25 ± 12.15	−0.17	−0.14	
	15ER	136.85 ± 6.39	122.89 ± 8.89^**^	88.70–156.10	124.95 ± 12.75	−0.10	0.04	
	16WH	168.50 ± 12.29	153.13 ± 8.75^*^	114.53–181.35	148.49 ± 13.22	−0.13	−0.32	
	16XN	143.90 ± 6.42	108.00 ± 5.06^***^	91.53–169.63	132.22 ± 13.02	−0.16	0.28	
	17WN	159.33 ± 12.09	102.30 ± 12.76^***^	96.29–181.88	134.44 ± 15.54	−0.03	−0.05	
BIH	15WH	42.63 ± 9.36	45.00 ± 9.86	12.50–71.50	40.76 ± 10.38	0.23	0.73	0.52
	15CD	44.58 ± 9.93	50.88 ± 11.63	3.00–58.00	26.76 ± 10.53	0.14	−0.45	
	15ER	43.05 ± 7.66	45.17 ± 6.74	10.70–71.80	40.50 ± 10.47	−0.04	0.62	
	16XN	82.29 ± 7.57	14.13 ± 6.48^***^	10.00–90.43	47.81 ± 16.15	0.11	−0.23	
	17WN	72.84 ± 12.07	24.00 ± 3.50^***^	15.00–102.00	43.17 ± 14.13	0.70	1.17	
SD	15WH	10.24 ± 2.17	9.06 ± 2.62^*^	6.34–13.89	9.86 ± 1.32	−0.04	0.23	0.55
	15CD	14.99 ± 2.15	11.85 ± 1.05^***^	7.93–18.42	13.09 ± 2.19	0.01	−0.43	
	15ER	10.76 ± 1.61	8.67 ± 1.59^***^	5.11–18.82	10.52 ± 1.71	1.07	4.18	
	16WH	13.07 ± 1.16	11.25 ± 1.80^***^	7.12–15.48	11.18 ± 1.27	−0.14	0.81	
	17WN	13.30 ± 2.21	8.42 ± 1.83^***^	6.73–15.94	11.31 ± 1.85	0.05	−0.46	
FT	15WH	144.2 ± 1.8	139.4 ± 2.4^*^	130.0–154.0	142.0 ± 3.0	−0.30	2.11	0.81
	15CD	148.2 ± 2.2	140.8 ± 1.6^**^	128.0–158.0	144.4 ± 4.8	−0.67	1.59	
	16WH	157.8 ± 2.4	141.2 ± 3.1^***^	121.3–162.4	146.7 ± 8.6	−0.77	0.23	
	16XN	62.6 ± 1.9	53.2 ± 2.4^***^	51.0–70.5	58.7 ± 3.9	0.70	0.14	
	17WN	74.4 ± 3	60.6 ± 2.3^***^	60.0–87.0	68.7 ± 5.3	0.93	1.28	

a *PH, plant height. BIH, branch initiation height. SD, stem diameter. FT, flowering time*.

b *15WH, Wuhan, 2015-2016; 15CD, Chengdu, 2015-2016; 15ER, Ezhou, 2015-2016; 16WH, Wuhan, 2016-2017; 16XN, Xining, 2016; 17WN, Weining, 2017*.

c The significance level between two parents:

* p ≤ 0.05;

** p ≤ 0.01;

*** *p ≤ 0.001*.

An ANOVA revealed that differences in genotypes, environments, and the interactions between genotype and environment were all highly significant (Table S2). The broad-sense heritabilities (*h*^2^) of PH, BIH, SD, and FT were calculated as 0.78, 0.52, 0.55, and 0.81, respectively (Table S2). Lower heritability of BIH and SD indicated that these two traits were influenced by environmental factors to a greater degree than were the other two traits. The Pearson's correlation coefficients between the four traits were detected in three representative environments (15WH, 15CD, and 17WN) (Table 2). As a result, all four traits were significantly and positively correlated with one another. For example, in the spring-type rapeseed area 17WN, PH was determined to be significantly positively correlated with BIH (*r* = 0.67, *P* < 0.001), SD (*r* = 0.76, *P* < 0.001), and FT (*r* = 0.61, *P* < 0.001); BIH was significantly positively correlated with both SD (*r* = 0.53) and FT (*r* = 0.68); and significant positive correlations were also observed between SD and FT (*r* = 0.54, *P* < 0.001) (Table 2).

**Table 2:** Correlations between PH, BIH, SD, and FT in 15WH, 15CD, and 17WN.

**Environment^a^**	**Trait^b^**	**PH**	**BIH**	**SD**
15WH	BIH	0.49^***^^c^		
	SD	0.66^***^	0.20^**^	
	FT	0.28^***^	0.53^***^	0.17^*^
15CD	BIH	0.32^***^		
	SD	0.52^***^	0.19^**^	
	FT	0.34^***^	0.32^***^	0.15^*^
17WN	BIH	0.67^***^		
	SD	0.76^***^	0.53^***^	
	FT	0.61^***^	0.68^***^	0.54^***^

a *15WH, Wuhan, 2015–2016; 15CD, Chengdu, 2015–2016; 17WN, Weining, 2017*.

b *PH, plant height; BIH, branch initiation height; SD, stem diameter; FT, flowering time*.

c The significance level:

* p ≤ 0.05;

** p ≤ 0.01;

*** *p ≤ 0.001*.

### Unconditional additive QTL mapping and meta-analysis

**Figure 2:**
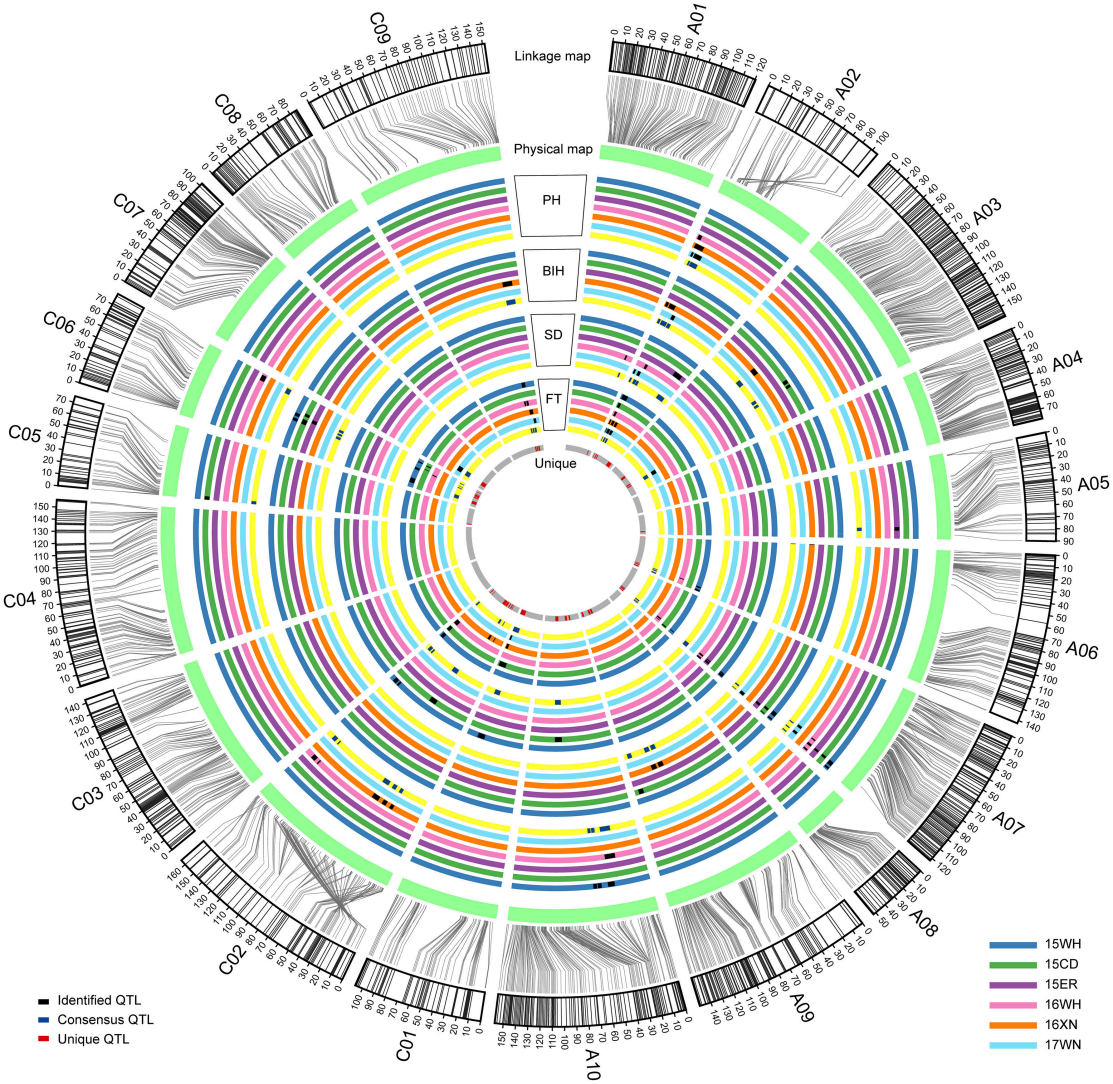
Process and results of the two-round QTL meta-analysis for PH, BIH, SD, and FT mapped on the chromosomes. All identified additive QTL were integrated into consensus QTL trait by trait, and then all consensus QTL collected from different traits were integrated into unique QTL. From the outermost circle to the second outer circle, the SNP markers on each chromosome were aligned to their positions in the physical map. From the third outer circle to the innermost, each color of circle represents one environment, except for the yellow and gray ones, which are used to describe the positions of consensus and unique QTL on chromosomes. In addition, black, blue and red blocks on circles represent the range of confidence intervals for identified, consensus, and unique QTL, respectively.

**Figure 3:**
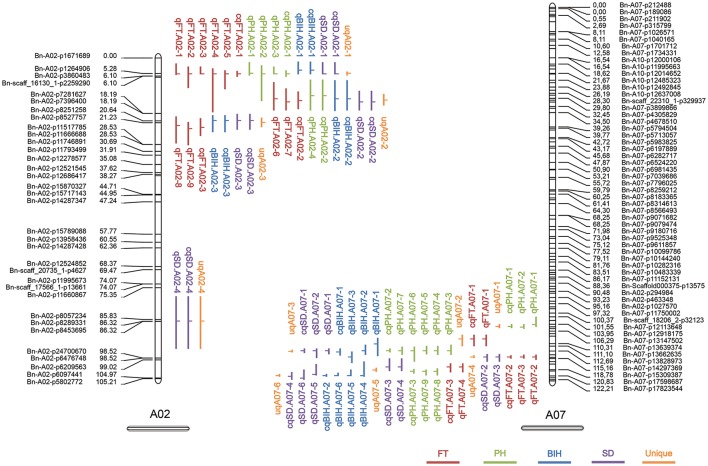
QTL clusters on chromosomes A02 and A07. The length of the vertical bar in the “⊣” and “⊢” symbol indicates the confidence interval of the QTL, and the position of the horizontal bar indicates the peak position of the QTL.

With the method of CIM, a total of 102 significant additive QTL associated with PH, BIH, SD, and FT were detected across 3 years (Table S3 and Figure 2). These QTL were primarily distributed on chromosomes A07 (25 QTL), A02 (20 QTL), C02 (13 QTL), and C06 (12 QTL), singly explained 2.88-32.60% of the phenotypic variance. They were subjected to the first round of QTL meta-analysis trait-by-trait, and resulted in 72 consensus QTL (Table S3). Of these, 19 consensus QTL were considered as major QTL, 11 (57.9%) of which were stably detected in at least two separate environments (Table 3). Notably, these major consensus QTL were all located on chromosomes A02 and A07, and organized into two major QTL clusters (Table 3 and Figure 3).

**Table 3:** Summary of the major consensus QTL and their corresponding identified QTL used for QTL meta-analysis.

**Trait**	**Major consensus QTL**					**Identified QTL**						
	**QTL^a^**	**Chr.^b^**	**Peak**	**CI^c^**	**PI (kb)^d^**	**QTL**	**LOD^e^**	**Peak**	**CI**	**Add.^f^**	***R*^2^ (%)**	**Env.^g^**
PH	cqPH.A02-1	A02	5.23	4.1–6.3	115–1,575	qPH.A02-1	4.16	5.01	1.6–6	3.42	6.07	16WH
						qPH.A02-2	11.72	5.31	2.6–14.3	6.19	20.09	16XN
						qPH.A02-3	8.03	5.31	3–5.6	6.60	15.50	17WN
	cqPH.A02-2	A02	12.41	7.2–17.4	1,575–4,331	qPH.A02-4	7.18	12.41	7.2–17.4	6.52	16.07	17WN
	cqPH.A07-1	A07	104.67	104–105.4	15,114–15,207	qPH.A07-1	2.61	104.01	101.5–104.8	6.41	4.86	17WN
						qPH.A07-2	10.05	104.81	104–105.5	6.05	16.76	16WH
	cqPH.A07-2	A07	112.71	112–113.4	15,704–15,944	qPH.A07-3	3.99	112.71	110.6–114	6.53	7.29	17WN
						qPH.A07-4	14.61	112.71	111.1–114	7.02	23.18	16WH
						qPH.A07-5	8.64	112.71	111.2–114	5.19	14.81	15CD
						qPH.A07-6	13.06	112.71	111.3–116.2	6.80	23.27	15WH
						qPH.A07-7	12.89	112.71	111.3–114	8.14	29.06	15ER
	cqPH.A07-3	A07	119.31	118.6–120.1	17,226–19,481	qPH.A07-8	13.38	119.31	118.5–120.8	6.93	23.74	15WH
						qPH.A07-9	13.87	119.31	118.8–120.8	6.89	22.19	16WH
BIH	cqBIH.A02-1	A02	4.01	1.4–5.5	115–1,280	qBIH.A02-1	12.56	4.01	1.4–5.5	7.92	21.99	16XN
	cqBIH.A02-2	A02	13.41	7.4–17.4	1,575–4,331	qBIH.A02-2	10.24	13.41	7.4–17.4	7.34	19.44	16XN
	cqBIH.A02-3	A02	20.61	18.9–24.6	5,233–8,233	qBIH.A02-3	10.81	20.61	18.9–24.6	6.43	19.83	17WN
	cqBIH.A07-1	A07	113.13	112–114.3	15,704–15,964	qBIH.A07-1	8.80	110.31	108.4–114.4	5.24	18.65	15WH
						qBIH.A07-2	6.15	112.71	110.6–114	4.19	11.41	15CD
						qBIH.A07-3	11.90	114.71	113–116.7	6.26	23.02	15ER
	cqBIH.A07-2	A07	120.81	120.3–121.3	18,917–22,002	qBIH.A07-4	3.11	120.81	116.2–121.3	3.60	5.20	17WN
						qBIH.A07-5	4.07	120.81	118.8–121	4.76	7.87	15WH
						qBIH.A07-6	8.88	120.81	119.9–121	4.85	16.01	15CD
SD	cqSD.A02-1	A02	5.31	1.6–5.6	115–1,280	qSD.A02-1	7.30	5.31	1.6–5.6	0.78	15.14	17WN
	cqSD.A07-1	A07	112.71	111.9–113.5	15,704–15,944	qSD.A07-1	7.32	112.71	112–113.9	0.89	17.48	15ER
						qSD.A07-2	7.83	112.71	112–115.2	0.99	12.30	16WH
	cqSD.A07-2	A07	114.71	113.7–116.2	15,943–16,684	qSD.A07-3	8.78	114.71	113.7–116.2	0.62	16.58	15WH
	cqSD.A07-3	A07	118.01	115.2–119.3	16,163–18,917	qSD.A07-4	6.14	118.01	115.2–119.3	0.86	15.53	15ER
	cqSD.A07-4	A07	120.97	119.7–122.2	18,917–22,002	qSD.A07-5	8.52	120.31	117–120.8	0.62	16.67	15WH
						qSD.A07-6	3.45	121.51	118.8–122.2	0.36	5.71	16WH
FT	cqFT.A02-1	A02	5.3	4.4–6.2	115–1,575	qFT.A02-1	4.52	5.31	1.9–5.6	1.35	6.03	16XN
						qFT.A02-2	6.01	5.31	2.9–9.9	1.42	7.95	15CD
						qFT.A02-3	5.62	5.31	3–5.5	2.06	7.42	17WN
						qFT.A02-4	8.57	5.31	3.6–18	0.99	10.39	15WH
						qFT.A02-5	20.98	5.31	4.2–10.5	4.74	27.03	16WH
	cqFT.A02-2	A02	14.02	11.2–16.9	1,575–4,331	qFT.A02-6	15.35	13.41	8.1–17.4	2.93	29.18	17WN
						qFT.A02-7	16.90	14.41	10.1–17.4	2.27	32.60	16XN
	cqFT.A07-2	A07	114.7	114.2–115.2	15,954–16,684	qFT.A07-2	20.57	114.71	114–115.7	1.93	28.81	15WH
						qFT.A07-3	13.76	114.71	114–115.3	4.09	16.21	16WH
	cqFT.A07-3	A07	118.01	116.8–119.8	16,171–18,917	qFT.A07-4	18.40	118.01	116.8–119.8	3.08	28.13	15CD

a *QTL underline indicate the novel major QTL that were not detected since the release of B.napus 60 K SNP array*.

b *Chromosome*.

c *Confidence interval*.

d *Physical interval*.

e *Logarithm of odds*.

f *Phenotypic variation explained by additive effect*.

g *Environment*.

For PH, 25 additive QTL were detected and account for 3.40–29.06% of the phenotypic variation. These QTL were integrated into 17 consensus QTL, with five QTL (cqPH.A02-1, cqPH.A02-2, cqPH.A07-1, cqPH.A07-2, and cqPH.A07-3) considered to be major QTL (Table S3 and Table 3). Of these, cqPH.A07-2 was repeatedly detected in five environments, and accounted for a mean PVE of 19.52%, with the highest PVE (29.06%) detected in 15ER (Table 3).

For BIH, 22 additive QTL were detected, with a PVE of 3.86–23.02%. These QTL were integrated into 16 consensus QTL, with five QTL (cqBIH.A02-1, cqBIH.A02-2, cqBIH.A02-3, cqBIH.A07-1, and cqBIH.A07-2) considered to be major QTL (Table S3 and Table 3). Two QTL (cqBIH.A07-1 and cqBIH.A07-2), which were detected in three of the five environments, accounted for 17.69 and 9.69% of mean PVE, respectively (Table 3).

For SD, 16 additive QTL with PVE ranging from 4.10 to 17.48% were integrated into 14 consensus QTL, and only two QTL (cqSD.A07-1 and cqSD.A07-4) were repeatedly identified in two environments (Table S3 and Table 3). This high proportion (87.5%) of environment-specific QTL suggests that SD is a complex trait and is greatly influenced by the environment, which was further reflected in its relatively low heritability (*h*^2^ = 0.55) (Table 1).

For FT, 39 additive QTL were detected, with PVE ranging from 2.84 to 32.60%. These QTL were integrated into 25 consensus QTL, with four consensus QTL (cqFT.A02-1, cqFT.A02-2, cqFT.A07-2, and cqFT.A07-3) considered to be major QTL (Table 3 and Table S3). One of the major QTL, cqFT.A02-1, was stably expressed in all five environments (mean PVE = 11.76%; LOD: 4.52–20.98), whereas cqFT.A02-2 was detected only in spring-type rapeseed areas 17WN and 16XN (mean PVE = 30.89%; LOD: 15.35–16.90), and cqFT.A07-2 was detected only in winter-type rapeseed areas 15 and 16 WH (mean PVE = 22.51%; LOD: 13.76–20.57). In addition, cqFT.A07-3 was an environment-specific QTL, although it explained 28.13% of the phenotypic variance in 15CD (Table 3).

To dissect the overlapping consensus QTL for the different traits, the second round of QTL meta-analysis was performed. In this round, all consensus QTL were integrated into 49 unique QTL, 15 (30.6%) of which controlled at least two traits (Table S4). Among these, two unique QTL (uqA02-1 and uqA02-2) located at the intervals of 4.51–5.79 and 12–15.69 cM on A02, respectively, were associated with all four traits, and two other unique QTL (uqA07-3 and uqA07-5) located at the intervals of 112.30–113.26 and 118.30–119.58 cM on A07, respectively, were associated with three of the four traits (Table 4).

**Table 4:** Unique QTL involved in more than two traits located on chromosomes A02 and A07.

**Unique QTL**				**Consensus QTL^c^**			
	**Chr.^a^**	**Peak**	**CI^b^**		**Trait**	**Peak**	**CI**
uqA02-1	A02	5.15	4.51–5.79	**cqSD.A02-1**	SD	5.31	1.6–5.6
				**cqFT.A02-1**	FT	5.3	4.37–6.24
				**cqBIH.A02-1**	BIH	4.01	1.4–5.5
				**cqPH.A02-1**	PH	5.23	4.13–6.33
uqA02-2	A02	13.85	12–15.69	**cqPH.A02-2**	PH	12.41	7.2–17.39
				**cqFT.A02-2**	FT	14.02	11.15–16.89
				cqSD.A02-2	SD	14.41	11–17.5
				**cqBIH.A02-2**	BIH	13.41	7.4–17.39
uqA02-3	A02	21.28	19.68–22.87	cqSD.A02-3	SD	20.61	18.5–23.7
				cqFT.A02-3	FT	22.78	19.9–25.65
				**cqBIH.A02-3**	BIH	20.61	18.89–24.6
uqA07-3	A07	112.78	112.30–113.26	**cqBIH.A07-1**	BIH	113.13	111.97–114.28
				**cqSD.A07-1**	SD	112.71	111.89–113.52
				**cqPH.A07-2**	PH	112.71	112.01–113.4
uqA07-4	A07	114.70	114.22–115.17	**cqSD.A07-2**	SD	114.71	113.7–116.2
				**cqFT.A07-2**	FT	114.7	114.19–115.22
uqA07-5	A07	118.94	118.30–119.58	**cqSD.A07-3**	SD	118.01	115.2–119.3
				**cqPH.A07-3**	PH	119.31	118.55–120.06
				**cqFT.A07-3**	FT	118.01	116.8–119.8
uqA07-6	A07	120.83	120.37–121.28	**cqBIH.A07-2**	BIH	120.81	120.32–121.29
				**cqSD.A07-4**	SD	120.97	119.7–122.24

a *Chromosome*.

b *Confidence interval*.

c *Major consensus QTL are in bold font. Consensus QTL explained more than 15% of the phenotypic variation in one environment or with the phenotypic variation more than 10% in two or more environments were defined as major QTL*.

### Conditional QTL mapping

Conditional QTL mapping was performed to reveal the additional additive QTL that could not be detected by unconditional QTL mapping methods. In total, 194 QTL were identified by conditional QTL mapping, including 63, 36, 24, and 71 QTL for PH, BIH, SD, and FT, respectively (Table S5). Of these, 99 QTL were also detected by unconditional QTL mapping, whereas the remaining 95 QTL could only be detected by conditional QTL mapping (Table S6). For instance, cdqFT|PH.A07-2, a QTL located on 114.7 cM of chromosome A07 (PVE = 17.33%), was detected by conditional QTL mapping in the environment 15CD. This QTL could not be detected by unconditional QTL mapping in 15CD, although it could be repeatedly detected in 15 and 16 WH (Tables S3, S6).

Conditional QTL mapping was also used to evaluate possible genetic relationships between PH, BIH, SD, and FT. Unconditional QTL mapping revealed the presentence of two major QTL clusters on chromosomes A02 and A07, including seven unique QTL that controlled more than two traits (Figure 3 and Table 4). Conditional QTL mapping was performed for preliminary discernment of whether unique QTL were due to one allele with pleiotropic effects or by multiple tightly linked but independent alleles (Li et al., 2014; Zhang et al., 2016). As a result, different unique QTL contributed differentially to the genetic basis of the correlated traits (Table S7). For instance, uqA02-1 was associated with all four traits; for FT, this QTL was detected in all five environments by unconditional QTL mapping, and when FT was conditioned on PH, BIH, and SD in conditional QTL mapping, it could also be detected in most of the environments, whereas for PH, BIH, and SD, conditional analysis failed to detect the QTL detected by unconditional analysis, suggesting that this unique QTL (i.e., uqA02-1) might contain two closely linked alleles, one specially for FT, and the other pleiotropic for PH, BIH, and SD (Table S7). Two other unique QTL on A02, uqA02-2, and uqA02-3, each showed signs of pleiotropy rather than tight linkage as the underlying genetic basis for the related traits, because the QTL could not be detected (or was only detected with strongly reduced effect when one of the traits was conditioned by one of the others) in unconditional QTL mapping (Table S7). For the unique QTL on A07, in general, tight linkage rather than pleiotropy might explain the co-localized QTL for the correlated traits, because most of the corresponding QTL could be detected in both conditional and unconditional mapping (Table S7). These results accorded with the interpretation that the median degree of pleiotropy is very limited (Wang et al., 2010; Wagner and Zhang, 2011).

### QTL by environment interactions mapping

To verify the additive QTL detected by the CIM algorithm and to estimate QEI, QEI mapping was performed using the phenotypic data from multiple environments, as well as their genotypes. Consequently, a total of 31 combined QTL associated with PH, BIH, SD, and FT were identified, using LOD thresholds of 5.92, 5.46, 5.41, and 5.37, respectively (Table 5). Of these, 23 QTL (74.1%) were also detected by CIM, including 11 major QTL (Table 5). Several of these combined QTL exhibited a strong QEI effect, such as IqBIH.C06-1, the PVE (A) of which was 0.04 and the PVE (A by E) was 5.52. In construct, others displayed weak QEI, such as IqPH.A07-1, with PVE (A) and PVE (A by E) values of 10.32 and 3.15, respectively. In general, the major QTL exhibited weaker QEI effects than did minor QTL. Among the traits PH, BIH, SD, and FT, four major QTL associated with BIH displayed strong QEI effects, indicating that BIH is highly influenced by environmental factors (Table 5).

**Table 5:** Summary of the combined QTL detected by QEI mapping.

**Trait**	**Combined QTL^a^**	**Chr.^b^**	**Pos.^c^**	**CI^d^**	**LOD^e^**	**LOD (A)^f^**	**LOD (AbyE)^g^**	**PVE^h^**	**PVE (A)^i^**	**PVE (AbyE)^j^**	**Add^k^**	**Consensus QTL^l^**
FT	**IqFT.A02-1**	**A02**	**5**	**4.5–5.5**	**56.68**	**54.13**	**2.55**	**39.33**	**26.43**	**12.90**	**2.01**	**cqFT.A02-1**
	IqFT.A02-2	A02	21	18.5–21.5	11.44	2.48	8.96	4.33	1.00	3.34	0.38	cqFT.A02-3
	IqFT.A03-1	A03	134	132.5–135.5	5.70	4.71	0.99	2.17	1.86	0.32	−0.51	
	IqFT.A06-1	A06	141	137.5–142	6.89	3.09	3.80	1.63	1.22	0.41	−0.42	cqFT.A06-3
	IqFT.A07-1	A07	55	53.5–55.5	5.38	3.84	1.55	4.00	1.52	2.48	0.50	
	**IqFT.A07-2**	**A07**	**114**	**113.5–114.5**	**54.88**	**27.51**	**27.36**	**22.01**	**12.05**	**9.97**	**1.40**	**cqFT.A07-2**
	IqFT.C01-1	C01	65	64.5–65.5	6.67	2.49	4.18	3.81	1.00	2.81	0.38	
	IqFT.C02-1	C02	34	33.5–34.5	5.42	2.83	2.59	3.84	1.10	2.73	0.41	cqFT.C02-1
	IqFT.C02-2	C02	47	46.5–47.5	5.94	2.04	3.90	1.75	0.83	0.92	0.35	cqFT.C02-2
	IqFT.C02-3	C02	159	156.5–159.5	15.92	12.51	3.40	7.47	5.11	2.36	−0.85	cqFT.C02-3
	IqFT.C03-1	C03	49	46.5–49.5	7.00	2.32	4.68	2.31	0.92	1.39	0.36	
	IqFT.C06-1	C06	70	69.5–70.5	16.53	3.65	12.88	3.95	1.48	2.47	−0.50	cqFT.C06-4
	IqFT.C09-1	C09	123	121.5–123.5	9.20	7.33	1.87	5.14	2.92	2.21	−0.66	cqFT.C09-2
PH	**IqPH.A02-1**	**A02**	**5**	**4.5–5.5**	**23.77**	**18.46**	**5.31**	**12.67**	**8.61**	**4.06**	**3.03**	**cqPH.A02-1**
	**IqPH.A07-1**	**A07**	**113**	**111.5–113.5**	**29.91**	**22.06**	**7.85**	**13.47**	**10.32**	**3.15**	**3.39**	**cqPH.A07-2**
	**IqPH.A07-2**	**A07**	**115**	**114.5–115.5**	**12.91**	**5.34**	**7.57**	**6.47**	**2.44**	**4.03**	**1.67**	**cqPH.A07-3**
	IqPH.A10-1	A10	54	51.5–54.5	6.88	4.93	1.96	2.84	2.24	0.60	1.49	cqPH.A10-3
	IqPH.C02-1	C02	48	47.5–48.5	6.26	4.52	1.74	2.83	2.05	0.78	1.46	cqPH.C02-2
	IqPH.C02-2	C02	154	150.5–154.5	8.02	3.48	4.54	3.51	1.58	1.94	1.25	cqPH.C02-5
	IqPH.C05-1	C05	0	0–0.5	7.03	4.55	2.48	2.87	2.06	0.82	−1.43	cqPH.C05-1
BIH	**IqBIH.A02-1**	**A02**	**5**	**2.5–5.5**	**10.89**	**5.53**	**5.36**	**19.77**	**5.53**	**14.24**	**1.66**	**cqBIH.A02-1**
	**IqBIH.A02-2**	**A02**	**20**	**18.5–21.5**	**10.06**	**3.70**	**6.37**	**13.80**	**3.66**	**10.14**	**1.30**	**cqBIH.A02-3**
	IqBIH.A07-1	A07	111	110.5–111.5	12.95	2.97	9.97	10.61	2.98	7.63	1.23	
	**IqBIH.A07-2**	**A07**	**114**	**113.5–114.5**	**15.08**	**3.47**	**11.61**	**11.24**	**3.50**	**7.73**	**1.36**	**cqBIH.A07-1**
	**IqBIH.A07-3**	**A07**	**120**	**119.5–120.5**	**9.66**	**3.00**	**6.66**	**7.80**	**2.87**	**4.93**	**1.23**	**cqBIH.A07-2**
	IqBIH.C01-1	C01	105	104.5–106	6.20	4.87	1.34	5.56	4.84	0.72	1.49	
	IqBIH.C03-1	C03	138	137.5–138.5	5.86	4.58	1.28	6.04	4.61	1.43	1.46	
	IqBIH.C06-1	C06	70	69.5–70.5	6.24	0.04	6.20	5.56	0.04	5.52	−0.15	
SD	**IqSD.A02-1**	**A02**	**5**	**1.5–5.5**	**7.45**	**3.89**	**3.56**	**7.46**	**3.37**	**4.09**	**0.20**	**cqSD.A02-1**
	IqSD.A02-2	A02	21	18.5–21.5	8.79	3.88	4.90	4.82	3.37	1.45	0.20	cqSD.A02-3
	**IqSD.A07-1**	**A07**	**113**	**111.5–113.5**	**11.86**	**6.90**	**4.96**	**8.55**	**5.99**	**2.56**	**0.28**	**cqSD.A07-1**

a *Combined QTL is the QTL detected by QEI mapping with ICIM algorithm. The corresponding major consensus QTL detected by CIM algorithm are in bold font*.

b *Chromosome*.

c *Chromosomal position (cM) of the peak*.

d *Confidence interval*.

e *LOD sore for additive and QEI effect*.

f *LOD score for additive effect*.

g *LOD score for QEI effect*.

h *Phenotypic variation explained by additive and QEI effect*.

i *Phenotypic variation explained by additive effect*.

j *Phenotypic variation explained by QEI effect*.

k *Estimated average additive effect of the QTL*.

l *The corresponding major consensus QTL detected by CIM algorithm*.

### Candidate genes mining

The available reference genome of *B. napus* and the functional annotation of the *A. thaliana* genome were exploited to identify candidate genes (Chalhoub et al., 2014). Notably, 21, 16, 26, and 45 candidate genes for PH, BIH, SD, and FT were identified in major consensus QTL regions, respectively (Table S8). For PH, candidate genes consisted mainly of three kinds of genes associated with phytohormones (GA, BR, and IAA), flower development, and phosphate ion transmembrane transport, such as *GASA4, GNL, LEP, FT, TCP, BAK1, CYP724A1, ARF8*, and *APT2*. For BIH, several candidate genes involved in cell differentiation, flower development and the gibberellic acid mediated signaling pathway were identified, including *LOF2, JGL, FT*, T*CP1, GA20OX4*, and *RGA2*. For SD, the candidate genes were genes primarily involved in cellular developmental processes, the gibberellic acid mediated signaling pathway, and plant-type cell wall organization, such as *MYB, AGP4, NAS2, KNU, WUS, KAN4, HY5, GASA4, UGD3*, and *EXPA23*. For FT, most of the candidate genes were related to light signaling (such as *BNQ3, COL3, HY5, ARR3*, and *TNY*), photoperiod (such as *AAT2, PRR9, SRR1, VEL1, CDF5*, and *MBD9*), flower development (such as *FT, NAP, MMP, SVP, VGT1*, and *SEP3*), and vernalization (such as *KIN2, ZAT12, VIP2, NUA*, and *CSTF64*) (Table S8).

Several of the candidate genes exhibited some degree of pleiotropy. For example, *BnaA02g00370, BnaA02g00920, BnaA02g01670*, and *BnaA02g02510*, which are orthologs of *Arabidopsis FLC, HY5, FY*, and *PRE2*, respectively, were found to be associated with both FT and SD. Notably, *BnaA02g02560*, an ortholog of *Arabidopsis GASA4*, was suggested as a candidate gene controlling PH, SD, and FT. One candidate gene, *BnaA02g08490*, an ortholog of *Arabidopsis GNL*, was determined to be associated with PH, BIH, and FT (Table S8).

## Discussion

### QTL clusters and environment-specific QTL

We previously developed one DH population from a cross between an alien introgression line Y689 and *B. napus* cv. Westar, designed as YW-DH population (Shen et al., 2018). The introgression was characterized by its plant architecture of compressed branches, and enhanced lignification of stems and other organs. The trait of the more intensive lignification of organs was caused by the introgression of the genetic element from *C. bursa*-*pastoris*, which likely resulted in the taller plant height and longer growth period of Y689 than the recipient cultivar. Consequently, some novel and major QTL for branch angle were detected using the YW-DH population (Shen et al., 2018).

In the present study, the YW-DH population was utilized to detect the QTL associated with four significantly positively correlated traits (PH, BIH, SD, and FT). A total of 19 major QTL were identified and organized into two QTL clusters located within 1.4–24.6 cM (with a physical interval of 0.1–8.2 Mb) on A02 and 104.0–122.2 cM (with a physical interval of 15.1–22.0 Mb) on A07 (Table 4). In general, the QTL cluster on A02 was stably detected in spring-type rapeseed growing areas, whereas the QTL cluster on A07 was stably detected in semi-winter-type growing areas (Figure 2 and Table 3). For example, cqFT.A02-2 on A02 was repeatedly detected in the spring-type areas 16XN and 17WN, with a high mean PVE of 30.89%, but was not detected in semi-winter-type areas. Another QTL on A07, cqBIH.A07-1, was repeatedly detected in the semi-winter-type areas 15WH, 15CD, and 15ER, with a mean PVE of 17.69%, but was not detected in spring-type areas (Table 3). These results were explained by QEI mapping, in which cqFT.A02-2 exhibited such a strong QEI effect that it could not be detected any more, and cqBIH.A07-1 had both lower total PVE and a higher PVE (A by E) (Table 5). Particularly, some QTL were repeatedly detected in most of the environments (such as cqFT.A02-1 and cqPH.A07-2), with lower PVE (A by E) in QEI mapping (Tables 3, 5). These findings will facilitate the utilization of appropriate QTL to match local growing conditions.

### Pleiotropy or tight linkage of the co-localized QTL

Co-localized QTL or pleiotropic genes are of great significance for breeders and geneticists, for assessing the effects of selective genetic improvement programs, or for understanding pathways of genetic activity (Hill and Zhang, 2012). Co-localized QTL for PH and FT have been identified in many crops, including rice (Wei et al., 2010; Cai et al., 2014; Weng et al., 2014; Zhou et al., 2016), maize (Durand et al., 2012) and barley (Wang et al., 2014). In rice, *Ghd7*, encoding a CCT domain protein, played pleiotropic roles for plant height, heading date, grains per panicle, and environmental response (Xue et al., 2008; Weng et al., 2014). Subsequently, several other genes or major QTL that had pleiotropic effects on plant height and heading date (flowering time) were cloned in rice, such as *DTH8/ Ghd8, Hd1*, and *Dlf1* (Wei et al., 2010; Yan et al., 2011; Zhang et al., 2012; Cai et al., 2014). At the QTL level, conditional analysis was performed to infer the causal relationships among the traits that shared QTL.

In this study, we revealed 95 QTL that could only be detected by conditional QTL mapping (Table S6), and the expression of these QTL may have been suppressed by the conditional traits. Excluding the effect of conditional traits facilitate to obtain more QTL, and know much information about the genetic basis of the target trait. In addition, conditional mapping was performed to estimate the unique QTL located on the two QTL clusters. However, the genetic basis (pleiotropy or tight linkage) of some unique QTL could only be estimated in spring-type (uqA02-2) or semi-winter-type (uqA07-4 and uqA07-5) rapeseed growing areas (Table S7), thus limiting comparisons of the results between conditional and unconditional QTL mapping for these QTL. Actually, the mathematical model-based method for evaluating the pleiotropy of a QTL is probably arbitrary (Wagner and Zhang, 2011), and further research should focus on increasing mapping resolution and performing gene functional analysis. In any case, conditional QTL mapping proved to be an efficient tool for preliminary assessments of the genetic basis of the correlated traits.

### Novel QTL and candidate genes for stem-related traits

Plant height and flowering time are important agronomic traits that have attracted the attention of many biologists and geneticists. After the release of the *B. napus* reference genome and the 60 K SNP array, it became more convenient to compare the mapping results obtained from different genetic backgrounds. Here, we compared our results with previous studies by anchoring a known marker physical position to the rapeseed reference genome. Because the information about markers in some studies was incomplete or their physical positions were ambiguous, we only compared our results to those that also used the 60 K SNP array to perform genotyping (Li et al., 2015, 2016; Schiessl et al., 2015; Xu et al., 2015; Sun et al., 2016; Wang et al., 2016; Luo et al., 2017; Zheng et al., 2017). As a result, only six of 17 PH QTL, two of 16 BIH QTL, and 13 of 25 FT QTL detected in this study have been reported (Table S9). Among the novel QTL, 11 were major QTL, including three PH QTL (cqPH.A02-2, cqPH.A07-1, and cqPH.A07-2), three BIH QTL (cqBIH.A02-1, cqBIH.A07-1, and cqBIH.A07-2) and five SD QTL (cqSD.A02-1, cqSD.A07-1, cqSD.A07-2, cqSD.A07-3, and cqSD.A07-4). Although all major FT QTL have been previously reported, none of the major SD QTL have been presented in previous studies, despite the strong correlation between these two traits in our YW-DH population (Table 2). The scarcity of research on the genetic architecture of SD in *B. napus* may be due to its relatively low heritability (Table 1). The results of our QTL mapping results (including the newly found major QTL) presented here provide valuable additional insight into the genetic architecture of the stem system, which may prove useful for rapeseed breeding.

We screened various genes as possible candidate genes for each trait based on GO annotations and data published in the scientific literature. Of these, some candidate genes were repeatedly identified for different traits, including *BnaA02g02560* and *BnaA02g08490*, orthologs of *Arabidopsis GASA4* and *GNL*, respectively. *GASA4* was a member of the *Arabidopsis GAST1*-like gene family, the over-expression of which could promote GA responses such as flowering and seed germination (Rubinovich and Weiss, 2010). The results of other studies suggest that *GASA4* might play various roles in cell wall elongation (Irshad et al., 2008). *GNL* is an important transcription target of the GA signaling pathway (Bi et al., 2005), as well as its paralogous GATA transcription factor *GNC* (*GATA, NITRATE-INDUCIBLE, CARBONMETABOLISM INVOLVED*), which act upstream from the flowering time regulator *SOC1* (*SUPPRESSOR-OFOVEREXPRESSION-OF-CONSTANS1*) to directly regulate FT (Richter et al., 2013a,b). As such, these two genes (*GASA4* and *GNL*) were both associated with GA, indicating the pleiotropic role of phytohormone in regulating correlated traits. Although our conditional QTL mapping results increased the likelihood that most co-localized QTL were caused by tight linkage mechanisms, it is possible that these potential candidate genes act upstream of the same pathway, such as GA signaling in this study (Fletcher et al., 2015).

### Alien introgression derived DH population contributes to QTL detection

Traditional QTL mapping and GWAS are two common approaches for studying the molecular mechanism of the four traits included in our analysis. Although seven of the eight studies used for comparison purposes relied on GWAS, we detected numerous novel major QTL in our YW-DH population, indicating that traditional QTL mapping remains a powerful tool for elucidating quantitative plant traits. The strong detection power of QTL mapping depends on a large population size and a high-density SNP map. Particularly, the parent Y689 used in this study was a *B. napus* introgression line derived from intertribal hybridization with *C. bursa*-*pastoris*, which increased the genetic divergence of the genomic structure and thus expanded the phenotypic diversity of the DH population.

In conclusion, using the excellent *B. napus* introgression line as one parent for the construction of DH population and a high-density SNP map, many powerful QTL associated with four positively and significantly correlated agronomic traits (PH, BIH, SD, and FT) were revealed. The major QTL were distributed primarily on chromosomes A02 and A07, and organized into two QTL clusters. As the increased degree of stem lignification in the alien introgression was associated with compressed branching, taller height, and longer growth period, it was worthwhile to further elucidate the genetic and developmental networks among these traits by using this population (Wei et al., 2017). These stem-related QTL should assist in the development of rapeseed varieties featuring plant architecture and lodging resistance traits that facilitate mechanical harvesting.

## Author contributions

ZL and YX conceived the experiment. YS performed the research. YS and EX contributed to phenotypic measurements. YS, XG, and YX contributed to data analysis. YS and ZL wrote the manuscript. All authors reviewed and approved this submission.

### Conflict of interest statement

The authors declare that the research was conducted in the absence of any commercial or financial relationships that could be construed as a potential conflict of interest. The reviewer FX declared a shared affiliation, with no collaboration, with several of the authors, YS, EX, XG, ZL, to the handling Editor.
